# Loss of the TGFβ-Activating Integrin αvβ8 on Dendritic Cells Protects Mice from Chronic Intestinal Parasitic Infection via Control of Type 2 Immunity

**DOI:** 10.1371/journal.ppat.1003675

**Published:** 2013-10-03

**Authors:** John J. Worthington, Joanna E. Klementowicz, Sayema Rahman, Beata I. Czajkowska, Catherine Smedley, Herman Waldmann, Tim Sparwasser, Richard K. Grencis, Mark A. Travis

**Affiliations:** 1 Manchester Immunology Group, Faculty of Life Sciences, University of Manchester, Manchester, United Kingdom; 2 Wellcome Trust Centre for Cell-Matrix Research, Faculty of Life Sciences, University of Manchester, Manchester, United Kingdom; 3 Manchester Collaborative Centre for Inflammation Research, University of Manchester, Manchester, United Kingdom; 4 Sir William Dunn School of Pathology, University of Oxford, Oxford, United Kingdom; 5 Institute of Infection Immunology, TWINCORE, Center for Experimental and Clinical Infection Research, Hannover, Germany; New York University, United States of America

## Abstract

Chronic intestinal parasite infection is a major global health problem, but mechanisms that promote chronicity are poorly understood. Here we describe a novel cellular and molecular pathway involved in the development of chronic intestinal parasite infection. We show that, early during development of chronic infection with the murine intestinal parasite *Trichuris muris*, TGFβ signalling in CD4+ T-cells is induced and that antibody-mediated inhibition of TGFβ function results in protection from infection. Mechanistically, we find that enhanced TGFβ signalling in CD4+ T-cells during infection involves expression of the TGFβ-activating integrin αvβ8 by dendritic cells (DCs), which we have previously shown is highly expressed by a subset of DCs in the intestine. Importantly, mice lacking integrin αvβ8 on DCs were completely resistant to chronic infection with *T. muris*, indicating an important functional role for integrin αvβ8-mediated TGFβ activation in promoting chronic infection. Protection from infection was dependent on CD4+ T-cells, but appeared independent of Foxp3+ Tregs. Instead, mice lacking integrin αvβ8 expression on DCs displayed an early increase in production of the protective type 2 cytokine IL-13 by CD4+ T-cells, and inhibition of this increase by crossing mice to IL-4 knockout mice restored parasite infection. Our results therefore provide novel insights into how type 2 immunity is controlled in the intestine, and may help contribute to development of new therapies aimed at promoting expulsion of gut helminths.

## Author Summary

Infection with intestinal parasitic worms is a major global health problem, with billions of people infected world-wide. Often these worms (known as helminths) develop a long-lasting chronic infection, due to failure of the host to mount the correct type of immune response that would normally expel the parasite. However, how the immune system is controlled leading to chronic helminth infection is not well understood. Here we identify a novel pathway of importance in the development of chronic helminth infection. Using a model parasite which infects mice, we find that a protein called transforming growth factor beta (TGFβ signals to T-cells early during the development of chronic infection and that blocking this signal protects mice from infection. We have also uncovered a key pathway and cell type that controls TGFβ function during development of chronic infection. When a protein called integrin αvβ8 is absent from dendritic cells of the immune system, TGFβ is no longer activated to signal to T-cells and mice are able to mount a protective (type 2) immune response resulting in worm expulsion. Our findings therefore provide new insights into how chronic infections develop and identify potential molecular targets for the prevention of chronic helminth infection.

## Introduction

Gastrointestinal parasitic helminth infections are extremely prevalent, affecting nearly one quarter of the world population. Development of chronic infection, defined as the presence of adult worms in the host, results in severe morbidity and health problems and has been heavily linked with promotion of poverty in affected regions [1]. Current treatments involve the use of anti-helminthic drugs to kill the parasite, but this does not prevent rapid re-infection with worms and encounters problems with drug resistance. As infections with these intestinal parasites are usually chronic, it is likely that helminths are able to influence the immune system to prevent their expulsion. Therefore, understanding the cellular and molecular pathways that regulate the immune response during helminth infection will be crucial in identifying novel therapeutic targets for these poorly managed infections.

A key cytokine that plays a multi-functional role in controlling immune responses is transforming growth factor beta (TGFβ) [2]. TGFβ can affect many different cell types, with data highlighting a crucial role for TGFβ in regulation of CD4+ T-cells, both dampening and promoting effector responses depending on the context of the immune response [3], [4]. Importantly, although many cells can produce TGFβ, it is always made as an inactive complex that must be activated to produce biological function [5]. Thus, activation of TGFβ is a key regulatory step in controlling the function of TGFβ in the immune system.

Given its importance in regulating diverse T-cell responses, it is not surprising that TGFβ plays a crucial role in the maintenance of immune homeostasis and prevention of autoimmunity. Thus, mice lacking TGFβ receptors in T-cells develop multi-organ inflammatory disease [6], [7] and lack of TGFβ production by T-cells results in autoimmunity and colitis [8]. Interestingly, recent data has implicated TGFβ-like molecules produced by helminths in regulating immune responses during parasite infection [9]. However, the function of TGFβ during helminth infection and how it is regulated to control immune responses to intestinal parasites is poorly understood.

Here we show that mice infected with the intestinal parasite *Trichuris muris*, a homologue of the human pathogen *Trichuris trichuria* [10], display enhanced TGFβ signalling in CD4+ T-cells early during infection and that antibody-mediated blockade of TGFβ significantly reduces worm burden during the development of a chronic infection.. We find that integrin αvβ8 expressed by dendritic cells (DCs), which we have previously shown to be a key pathway in activating TGFβ during intestinal homeostasis [11], [12], is required for early induction of TGFβ signalling in CD4+ T-cells during development of chronic helminth infection. Importantly, mice lacking integrin αvβ8 expression on DCs are completely protected from chronic infection, with this protection resulting from a specific early upregulation of a Th2-type immune response. Our results therefore provide novel insights into regulatory mechanisms of importance during chronic gastrointestinal parasite infection, and may help contribute to the development of new therapies aimed at promoting expulsion of helminth infection.

## Results

### TGFβ signalling in CD4+ T-cells is increased early during development of chronic *T. muris* infection and blocking TGFβ protects mice from infection

**Figure 1:**
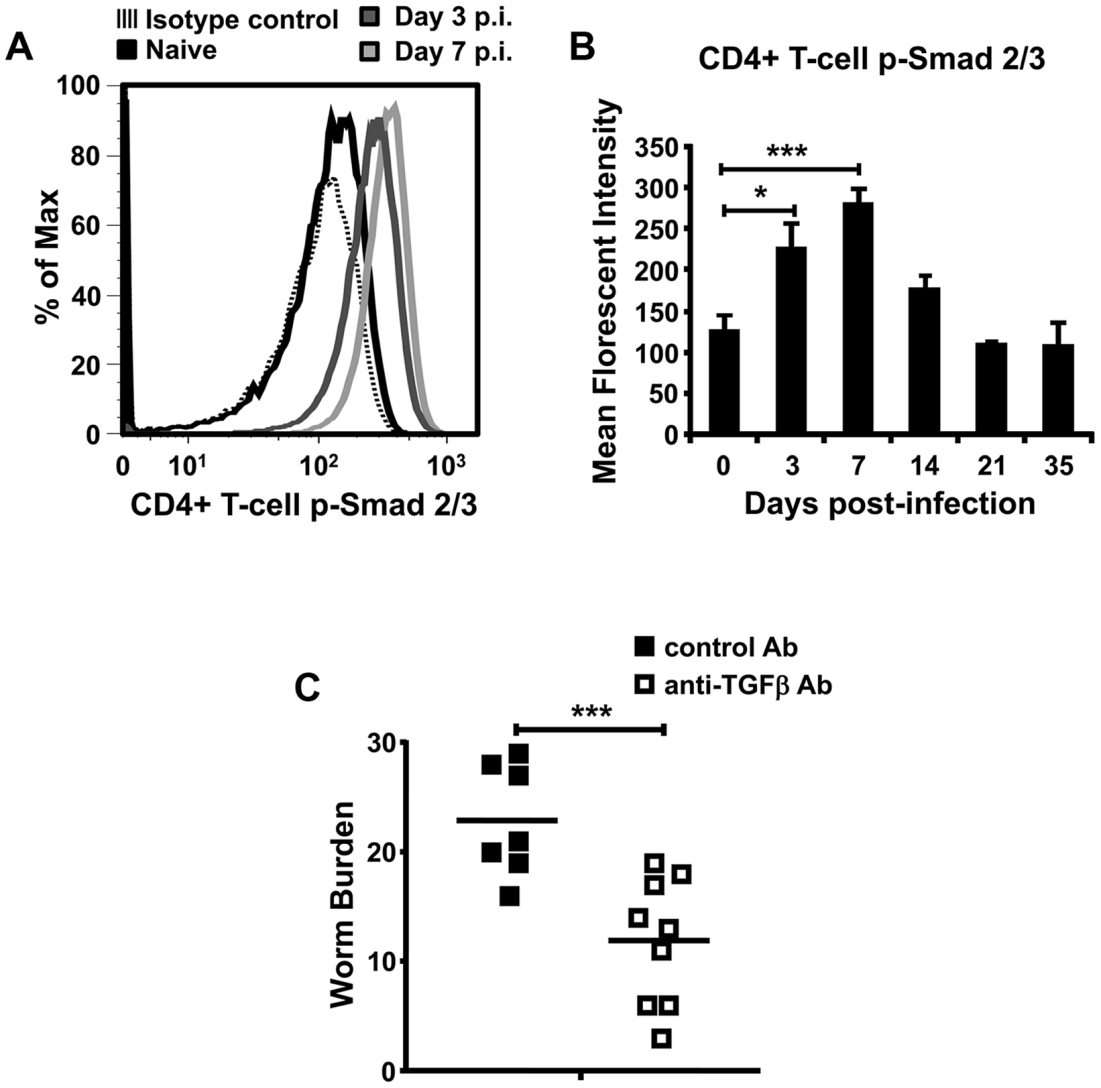
TGFβ is functionally important in the development of chronic *T. muris* infection. (*A*) and *(B)* Analysis of p-Smad 2/3 in CD4+ T-cells from mLN during development of a chronic *T. muris* infection in C57BL/6 mice. (*A*) Representative flow cytometry plots in naive mice and mice analysed 3 or 7 days post-infection (p.i.), (*B*) analysis of p-Smad 2/3 (MFI) in mLN CD4+ T-cells at different timepoints during infection. Data (n = 4–8 mice per group) are from up to three independent experiments performed. *(C)* Worm burdens from control and anti-TGFβ antibody (clone 1D11)-treated C57BL/6 mice analysed at day 21–23 p.i. after infection with a chronic dose of *T. muris* eggs. Data (n = 7–9 mice per group) are from two independent experiments performed. *, P<0.05; ***, P<0.005 via Kruskal–Wallis (B) and Student's *t*-test (C), error bars represent SE of means.

Development of a chronic parasite infection is believed to result from an inappropriate suppression of host immunity, although the exact molecular mechanisms governing these pathways remain unclear. Given the fundamental importance of CD4+ T-cells in regulating parasite infection and the key role for TGFβ in regulating many aspects of T-cell biology, we analysed TGFβ signalling in T-cells during development of a chronic infection with the helminth *Trichuris muris*. In C57BL/6 mice receiving 30 *T. muris* eggs, a dose shown previously to induce a chronic infection [13], we observed a specific increase in phosphorylation of Smad 2/3 (pSmad2/3) in mLN CD4+ T-cells, which is the initial signalling event triggered by engagement of TGFβ with its receptor [14]. This increase in TGFβ signalling was observed as early as day 3 post-infection, and was still evident at day 7 post-infection (Figure 1A and B), before returning to levels seen in uninfected mice by day 14 post-infection (Figure 1B). Similar early increases in CD4+ T-cell pSmad2/3 were also observed in cells taken from the lamina propria of the parasite's niche, the caecum and proximal colon (Figure S1 in Text S1). These data indicate that TGFβ signalling in CD4+ T-cells is an early hallmark of chronic *T. muris* infection.

To directly examine the functional importance of TGFβ in the development of a chronic *T. muris* infection, we injected C57BL/6 mice with a TGFβ function-blocking antibody before and during infection. Interestingly, mice receiving TGFβ function-blocking antibody were significantly protected from worm infection (Figure 1C). Thus, our data indicate that, during development of chronic infection, TGFβ plays an important role in promoting infection by the intestinal parasite *T. muris*.

### Expression of integrin αvβ8 by DCs propagates TGFβ signalling in CD4+ T-cells and development of chronic intestinal helminth infection

**Figure 2:**
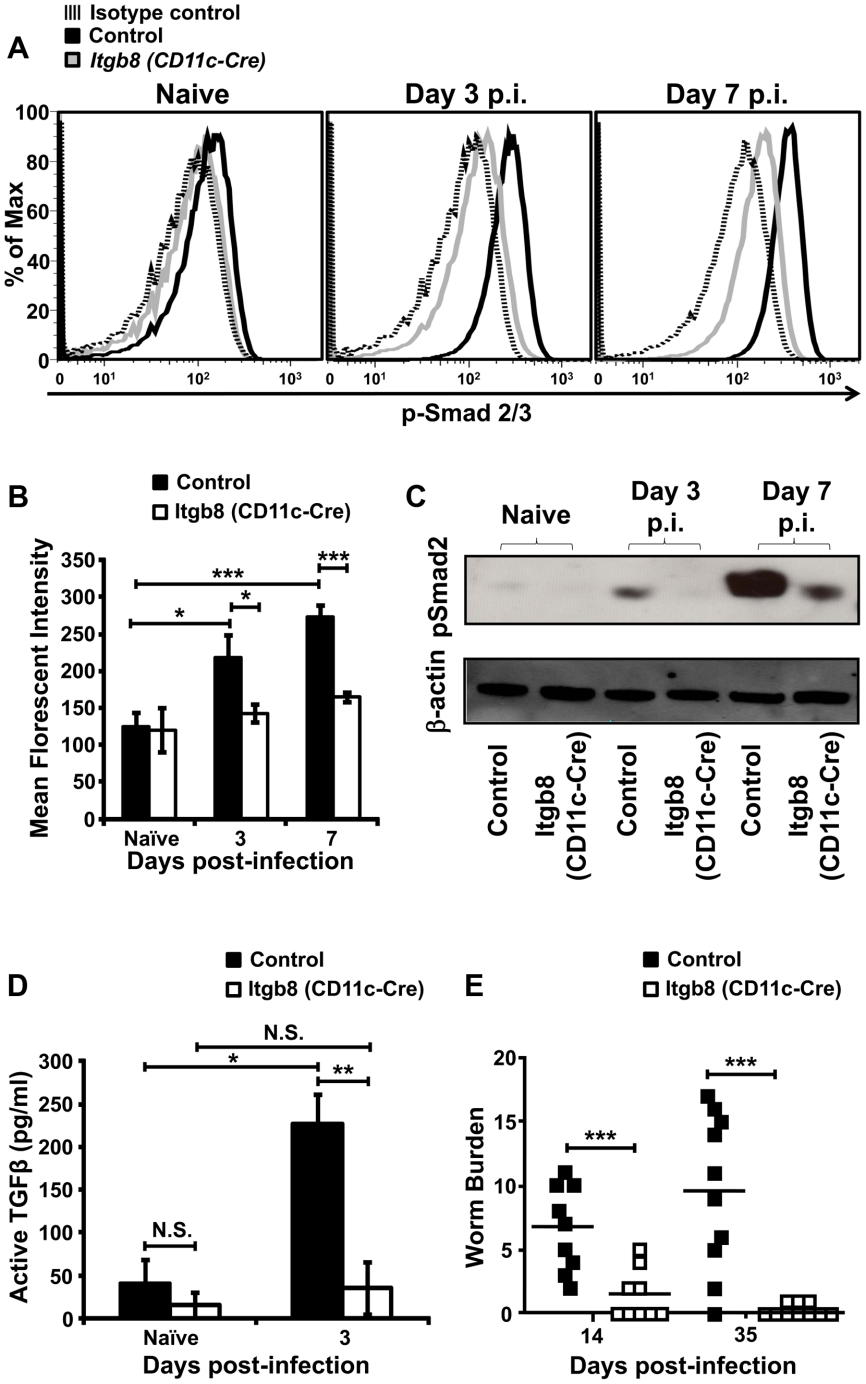
Mice lacking the TGFβ-activating integrin αvβ8 on DCs are protected from a chronic *T. muris* infection. (*A*) Representative flow cytometry plots and (*B*) average MFI values for p-Smad 2/3 staining in CD4+ T-cells from control and *Itgb8 (CD11c-Cre)* mice at different times after infection of mice with a chronic dose of *T. muris* eggs. Data (n = 5–8) are from three independent experiments performed. *(C)* Western blot analysis of p-Smad2/3 and β-actin in purified CD4+ T-cells from control and *Itgb8 (CD11c-Cre)* mice at different times during infection with a chronic dose of *T. muris*. Data representative of two independent experiments. (*D*) TGFβ activation by intestinal DCs isolated from control or *Itgb8 (CD11c-Cre)* mice, from naive mice or day 3 post-infection (p.i.) with a chronic dose of *T. muris* eggs, detected by co-culture with an active TGFβ reporter cell line [15]. Data (n = 3–4) are from three independent experiments performed. *(E)* Worm burdens from control and *Itgb8 (CD11c-Cre)* mice at day 14 and 35 p.i. with a chronic dose of *T. muris* eggs. Data (n = 9–10 mice per group) are from at least two independent experiments performed. *, P<0.05, ***, P<0.005 via Kruskal–Wallis (B) and Student's *t*-test (D and E) for the indicated comparisons between groups, error bars represent SE of means.

We next sought to determine the mechanisms responsible for enhanced TGFβ signalling and function during *T. muris* infection. One potential explanation for enhanced TGFβ signalling observed in CD4+ T-cells is enhanced activation of host latent TGFβ during infection. We have recently identified integrin αvβ8, expressed by DCs, as a key activator of latent TGFβ in the intestine during immune homeostasis [11], [12]. Thus, to determine the importance of this pathway in promoting TGFβ signalling in CD4+ T-cells during *T. muris* infection, we analysed T-cell responses in C57BL/6 control mice and mice lacking integrin αvβ8 on DCs (*Itgb8* (*CD11c-Cre*) mice) [11] during infection. Interestingly, the increase in TGFβ signalling observed in CD4+ T-cells early during *T.muris* infection was significantly reduced in *Itgb8* (*CD11c-Cre*) mice, with pSmad2/3 levels remaining similar to those observed in uninfected mice during the first week of infection (Figure 2A and B). This integrin αvβ8-dependent induction of Smad2/3 phosphorylation was confirmed by Western blot analysis for pSmad2/3 in purified CD4+ T-cells from infected mice (Figure 2C). In contrast, we did not observe any differences in pSmad2/3 induction in dendritic cells between control and *Itgb8* (*CD11c-Cre*) mice (Figure S2A and B in Text S1), indicating that the integrin αvβ8-mediated TGFβ activation does not trigger autocrine TGFβ signalling in DCs during early infection.

To directly test whether DCs produced enhanced levels of active TGFβ via expression of integrin αvβ8 during *T. muris* infection, we isolated DCs from control and *Itgb8* (*CD11c-Cre*) mice and measured their ability to activate TGFβ using an established active TGFβ reporter cell line [15]. Indeed, we observed an enhanced ability of intestinal DC activation to produce active TGFβ early during the development of chronic *T. muris* infection, which was completely absent in DCs lacking expression of integrin αvβ8 (Figure 2D). Thus, during development of chronic *T. muris* infection, enhanced TGFβ activation by integrin αvβ8 on DCs is important in triggering TGFβ signalling pathways in CD4+ T-cells.

To determine whether TGFβ activation by integrin αvβ8 on DCs was functionally important during development of chronic infection with *T. muris*, we analysed worm numbers in control and *Itgb8* (*CD11c-Cre*) mice infected with a chronic dose of *T. muris* eggs. Strikingly, *Itgb8* (*CD11c-Cre*) mice were completely protected from chronic infection by *T. muris* at day 35 post-infection, with mice showing protection as early as day 14 post-infection (Figure 2E). Indeed, protection from infection observed in *Itgb8* (*CD11c-Cre*) mice was even more pronounced than that observed using antibody-mediated blockade of TGFβ function (Figure 1C).

It has been reported that expression of CD11c-Cre may drive recombination in a subset of CD4+ CD11c^lo^ activated T-cells [16], and we have previously reported that integrin αvβ8 is expressed by CD4+ T-cells [11]. Thus, to test whether protection from infection in *Itgb8* (*CD11c-Cre*) mice could be due to deletion of the integrin in T-cell subsets, we infected mice lacking integrin αvβ8 on T-cells via expression of CD4-Cre (*Itgb8* (*CD4-Cre*) mice) [11]. In contrast to *Itgb8* (*CD11c-Cre*) mice, *Itgb8* (*CD4-Cre*) mice showed no protection from infection with *T.muris* (Figure S3A in Text S1) and showed an identical parasite-specific IgG2a/IgG1antibody bias which is associated with development of a chronic infection (Figure S3B in Text S1). Taken together, these data suggest that integrin αvβ8-mediated TGFβ activation by DCs is essential in the promotion of chronic *T. muris* infection.

### Protection from infection in *Itgb8* (*CD11c-Cre*) mice is driven by CD4+ T-cells but appears independent of Foxp3+ Tregs

**Figure 3:**
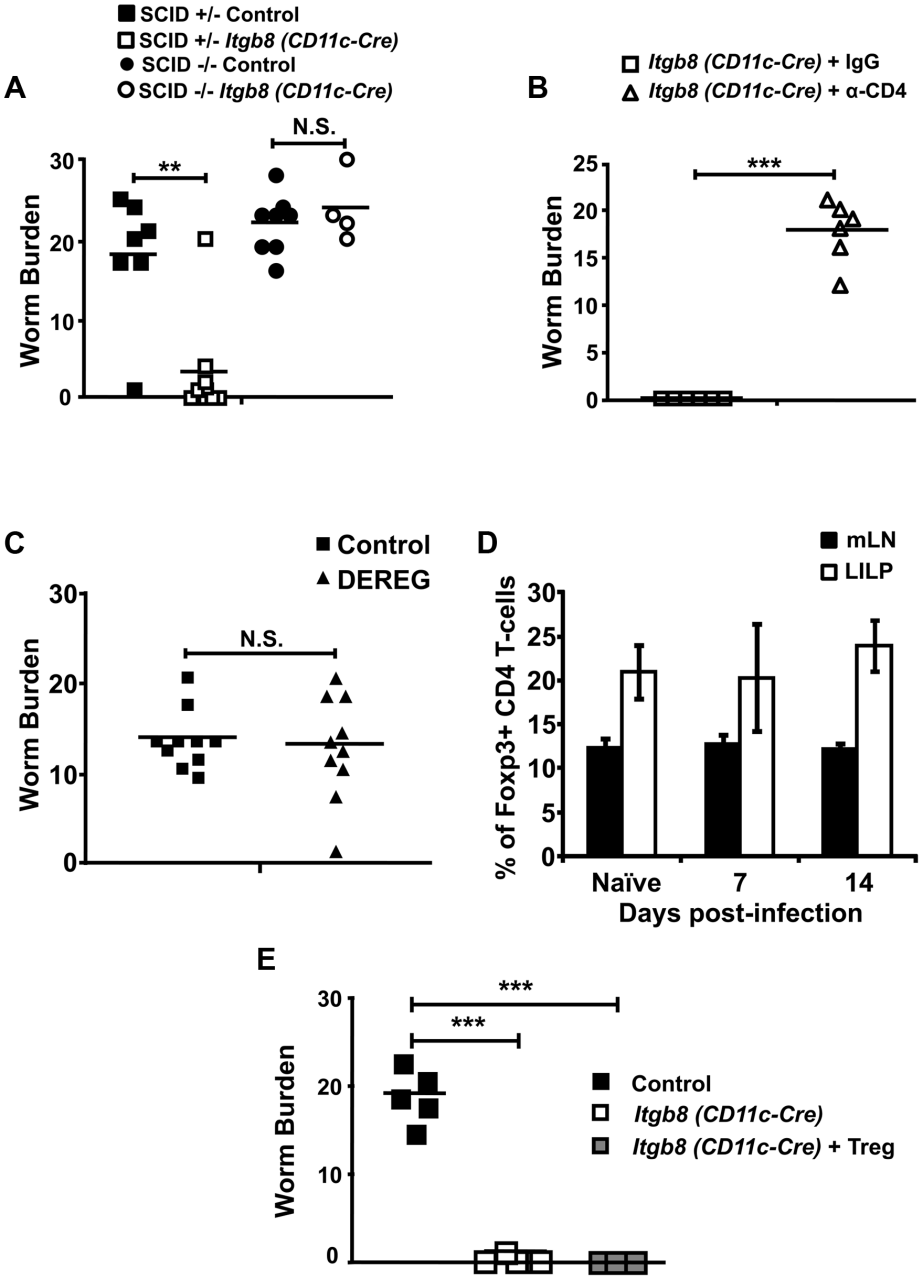
Protection from chronic *T. muris* infection in mice lacking the TGFβ-activating integrin αvβ8 on DCs is dependent on CD4+ T-cells, but does not involve Foxp3+ Tregs. (*A*) Worm burdens from control and *Itgb8 (CD11c-Cre)* mice crossed onto a SCID background analysed at day 32 post-infection (p.i.) with a chronic dose of *T.muris* eggs. Data (n = 4–9 mice per group) are from two independent experiments performed. (*B*) Worm burdens from *Itgb8 (CD11c-Cre)* mice infected with a chronic dose of *T.muris* eggs and treated with 2 mg of control IgG or anti-CD4 antibody (YTS191) analysed at day 17 p.i. Data (n = 6 mice per group) are from two independent experiments performed. (*C*) Control and DEREG mice were infected with a chronic dose of *T. muris* eggs, injected i.p. with 200 ng diphtheria toxin every 2 days (starting 2 days before infection) and worm burdens analysed at day 14 p.i. Data (n = 10 mice per group) are from two independent experiments performed. (*D*) Percentage of Foxp3+ Tregs of CD4 T-cells in mLN, IEL and LILP populations during *T. muris* infection as analysed by flow cytometry. Data (n = 7–11 mice per group) are from four independent experiments. (*E*) Caecal lamina propria worm burdens in control, *Itgb8 (CD11c-Cre)* and *Itgb8 (CD11c-Cre)* mice injected with 0.5×10^6^ GFP-Foxp3+ CD4 T-cells 3 days prior to infection. All mice received a chronic dose of *T. muris* and were analysed at day 14 p.i. for worm burden or day 3 p.i. for reconstitution. Data (n = 3–4 mice per group) are from three independent experiments. **, P<0.01; or ***, P<0.005, N.S., not significant via Kruskal–Wallis (D) and Student's *t*-test (A, B, C and E) for the indicated comparisons between groups, error bars represent SE of means.

We next sought to determine the mechanisms responsible for protection from infection in mice lacking the TGFβ-activating integrin αvβ8 on DCs. CD4+ T-cells are key in promoting expulsion of intestinal parasite infection, including *T. muris* [17], and TGFβ signalling is triggered in these cells early during infection (Figure 1A and B). However, recent evidence has proposed that novel innate lymphoid cells can play crucial roles in the expulsion of several parasite infections [18], [19], [20], [21]. Thus, to determine the function of a CD4+ T-cell response in the expulsion of *T. muris* observed in *Itgb8* (*CD11c-Cre*) mice, we first bred mice onto a C57BL/6 SCID background lacking all lymphocytes. In the absence of total lymphocytes, protection from infection was completely absent, with *Itgb8* (*CD11c-Cre*) SCID−/− mice showing similar susceptibility to infection as control mice (Figure 3A). To specifically test the role of CD4+ T-cells in protection from infection observed in *Itgb8* (*CD11c-Cre*) mice, we depleted CD4+ T-cells using an anti-CD4 antibody (Figure S4A in Text S1). Absence of CD4+ T-cells restored susceptibility to infection in *Itgb8* (*CD11c-Cre*) mice (Figure 3B). Taken together, these results indicate that protection from infection in the absence of integrin αvβ8 expression on DCs is not via a direct effect of innate lymphoid cells, but driven by a classical CD4+ T-cell response, although a role for innate cells in initial priming cannot be ruled out.

CD4+ Foxp3+ regulatory T-cells (Tregs) have been implicated in inhibiting immune responses to helminths [22] including some strains of *T. muris* [23]. Additionally, we have previously shown that integrin αvβ8-mediated TGFβ activation by specialised intestinal DCs is a crucial mechanism by which Foxp3+ Tregs are induced in the gut [12], and that *Itgb8* (*CD11c-Cre*) mice have reduced Foxp3+ Treg levels in their intestine [11]. Thus, one potential explanation for protection from infection in *Itgb8* (*CD11c-Cre*) mice is that there is a reduced Treg response induced during infection in these mice. To address this possibility, we first directly assessed the role of Foxp3+ Tregs during development of chronic *T. muris* infection by using the DEREG mouse model on a C57BL/6 background, which allows specific ablation of Foxp3+ Tregs by injection of diphtheria toxin [24]. Despite robust depletion of Foxp3+ Tregs (Figure S4B in Text S1) we did not see any enhanced ability of Foxp3+ Treg-depleted mice to expel worms (Figure 3C). In agreement with a lack of role for Foxp3+ Tregs in the development of chronic *T. muris* infection, we did not see any enhancement of Foxp3+ Treg levels during the course of infection (Figure 3D). Additionally, to directly assess whether reduced Foxp3+ Treg numbers *Itgb8* (*CD11c-Cre*) mice was responsible for protection from infection, we rescued Treg numbers by adoptively transferred Foxp3+ Tregs from GFP-Foxp3 mice [25] prior to infection. Despite enhancement of Treg numbers in *Itgb8* (*CD11c-Cre*) mice after adoptive transfer of GFP-Foxp3+ Tregs (Figure S5 in Text S1), *Itgb8* (*CD11c-Cre*) mice were still highly protected from development of a chronic infection (Figure 3E). Taken together, these data indicate that the protection from infection observed in *Itgb8* (*CD11c-Cre*) mice is driven by CD4+ T-cells, but independently of Foxp3+ Treg cells.

### *Itgb8* (*CD11c-Cre*) mice show a specific enhanced CD4+-mediated IL-13 response early after infection

**Figure 4:**
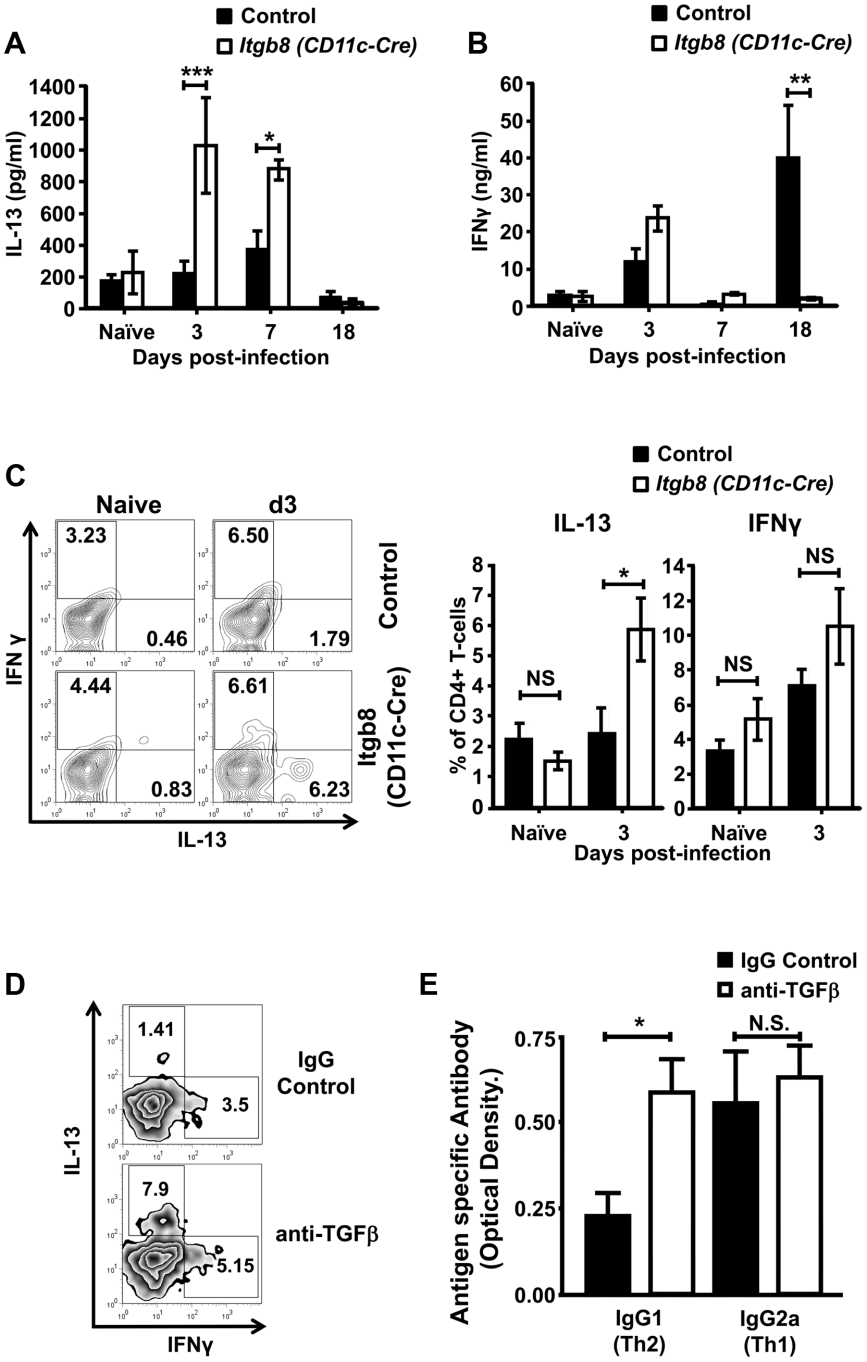
Mice lacking the TGFβ-activating integrin αvβ8 on DCs demonstrate an enhanced IL-13 production in LILP CD4+ T-cells. (*A*) IL-13 and (*B*) IFNγ cytokine levels from *T. muris* E/S antigen-stimulated mLN cells from control and *Itgb8 (CD11c-Cre)* mice at different time-points post-infection ( p.i.) with a chronic dose of *T. muris* eggs, determined via cytometric bead array/ELISA. Data (n = 5–6 mice per group) are from three or more independent experiments. (*C*) Analysis of intracellular IL-13 and IFNγ expression in CD4+ T-cells isolated from control and *Itgb8 (CD11c-Cre)* mice at day 3 p.i. with a chronic dose of *T. muris* eggs. Representative flow cytometry plots and mean data are displayed. Data (n = 5–6 mice per group mice per group) are from three independent experiments. (*D*) Representative flow cytometry plots for IL-13 and IFNγ from LILP CD4+ T-cells at day 3 p.i. and (*E*) Serum antigen specific IgG1 and IgG2a levels at day 21–23 p.i. from control and anti-TGFβ antibody-treated C57BL/6 mice infected with a chronic dose of *T. muris* eggs. Data (n = 7–9 mice per group) are from two independent experiments performed. *, P<0.05; **, P<0.01; or ***, P<0.005, N.S., not significant via Kruskal–Wallis (A and B) and Student's *t*-test (C and E) for the indicated comparisons between groups, error bars represent SE of means.

During development of a chronic infection with *T. muris*, mice develop a Th1-type immune response at the expense of a protective Th2-type response [13]. Thus, an alternative explanation for the expulsion of a normally chronic infection of *T. muris* by *Itgb8* (*CD11c-Cre*) mice is that, in the absence of early CD4+ T-cell TGFβ signalling, mice produce a Th2-type response instead of the usual non-protective Th1 response. To test this possibility, we analysed the production of Th1 and Th2 cytokines during infection in control and *Itgb8* (*CD11c-Cre*) mice. Strikingly, as early as 3 days post-infection, we observed a significant increase in production of the Th2 cytokine IL-13, which was still elevated at 7 days post-infection (Figure 4A). In contrast, although there was a slight enhancement of the Th1 cytokine IFNγ 3 days post-infection, this was not significantly different between control and *Itgb8* (*CD11c-Cre*) mice (Figure 4B). Control mice developed a marked enhancement in IFNγ production by day 18 post-infection, as expected during development of a chronic infection, and this was not observed in *Itgb8* (*CD11c-Cre*) mice (Figure 4B). Neither control nor *Itgb8* (*CD11c-Cre*) mice produced any detectable IL-4 at any tested timepoint post-infection, a cytokine previously shown to be involved in protection from *T. muris* infection (Figure S6 in Text S1 and data not shown).

We next investigated the cellular source of the early IL-13 production in *Itgb8* (*CD11c-Cre*) mice using flow cytometry. We observed a significant population of IL-13+ CD4+ T-cells within the intestinal lamina propria early during infection in *Itgb8* (*CD11c-Cre*) which was not apparent in control mice (Figure 4C). We also observed a slight increase in IFNγ+ lamina propria CD4+ T-cells in *Itgb8* (*CD11c-Cre*) mice early post-infection; however, these levels were not significantly different from those seen in control mice (Figure 4C). Interestingly, in mice treated with a TGFβ function-blocking antibody which resulted in protection from infection (Figure 1C), we observed a similar increase in CD4+ T-cell IL-13 production, with no difference in IFNγ production observed (Figure 4D). Furthermore, mice treated with TGFβ blocking antibody developed a skewed parasite-specific IgG1 response during infection, indicative of an enhanced type2 immune response (Figure 4E). Taken together, these data indicate that TGFβ activation by DC-expressed integrin αvβ8 is important in controlling IL-13 production in CD4+ T-cells early during development of chronic infection.

### Enhanced type 2 cytokine production by *Itgb8* (*CD11c-Cre*) mice is responsible for rapid *T. muris* expulsion

**Figure 5:**
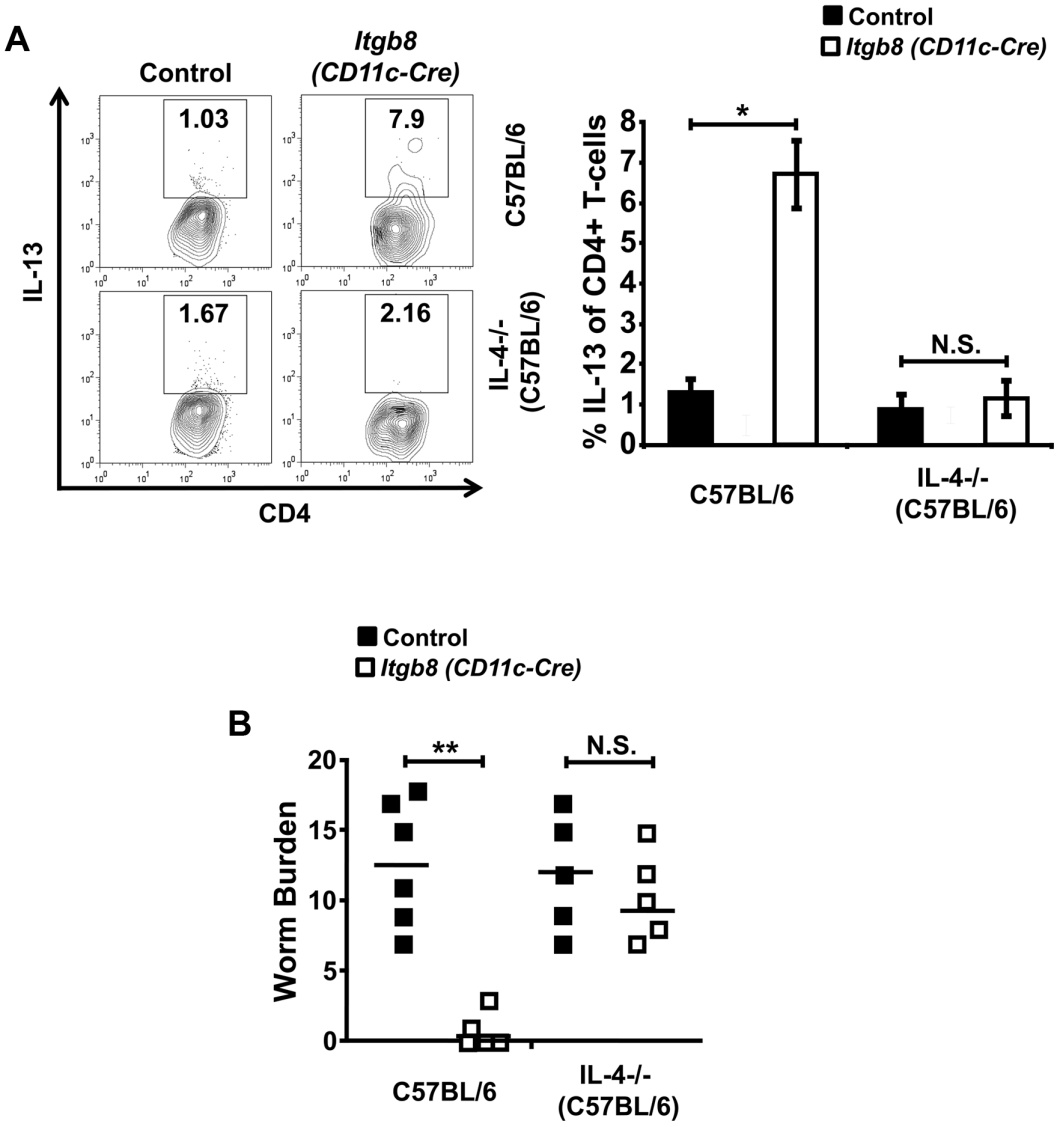
Ablation of enhanced IL-13 production by CD4+ T-cells in *Itgb8 (CD11c-Cre)* mice restores susceptibility to infection with *T. muris*. (*A*) IL-13 production by LILP CD4+ T-cells isolated day 3 post-infection (p.i.) with a chronic dose of *T. muris* eggs in IL-4−/− control and *Itgb8 (CD11c-Cre)* IL-4−/− mice. Representative flow cytometry plots and mean data are displayed. Data (n = 5–6 mice per group) are from two independent experiments. Data (n = 5–6) are from two independent experiments performed. (*B*) Worm burdens from IL-4−/− and *Itgb8 (CD11c-Cre)* IL-4−/− mice at day 14 p.i. with a chronic dose of *T. muris* eggs. Data (n = 5–6 mice per group) are from two independent experiments. **, P<0.01; N.S., not significant via Student's *t* –test for the indicated comparisons between groups, error bars represent SE of means.

To test whether the enhanced production of IL-13 early during infection was responsible for expulsion of a chronic *T. muris* infective dose, we crossed the *Itgb8 (CD11c-Cre)* mice with C57BL/6 IL-4 knockout mice, which have previously been shown to lack the ability to generate an IL-4/13-mediated Th2 response during *T. muris* infection [26]. As both control mice and *Itgb8 (CD11c-Cre)* mice did not display production of IL-4 early during *T. muris* infection (Figure S6 in Text S1), these mice allowed us to test the role of the enhanced IL-13 response seen early during infection in *Itgb8 (CD11c-Cre)* mice. As expected, *Itgb8* (*CD11c-Cre*)×IL-4−/− mice no longer demonstrated an early IL-13 production in the intestinal CD4+ T-cells (Figure 5A). Strikingly, *Itgb8* (*CD11c-Cre*)×IL-4−/− mice were completely susceptible to infection, with parasite burdens comparable to those seen in control mice (Figure 5B). Taken together, these data indicate that lack of the TGFβ-activating integrin αvβ8 on DCs results in a heightened CD4+ T-cell Th2 immune response during *T. muris* infection which is responsible for rapid parasite expulsion.

## Discussion

Infection with intestinal helminths can result in either expulsion or development of chronic infection, often depending on the type of CD4+ T-cell response generated. Generally, a chronic infection results when inappropriate Th1 cytokine production occurs, as opposed to an inability of CD4+ T-cells to mount a response. Expulsion of the parasite relies on the production of Th2 cytokines, in particular IL-13 which drives a combination of cytokine-mediated expulsion mechanisms such as increased epithelial cell turnover in the intestine [27], enhanced mucus production [28] and increased production of RELM-β [29]. Our data now demonstrate an essential role for TGFβ and the TGFβ-activating integrin αvβ8 expressed by DCs in promoting chronic intestinal parasite infection, using *T. muris*, a mouse model of the prevalent human parasite *Trichuris trichuria*. We observed that TGFβ signalling in CD4+ T-cells is triggered early during *T. muris* infection, and antibody-mediated blockade of TGFβ function significantly protects mice from infection. Mechanistically, we find that enhanced TGFβ signalling in T-cells during infection occurs via expression of the TGFβ-activating integrin αvβ8 on DCs and that lack of this integrin on DCs completely protects mice from infection due to an enhanced protective Th2 response. We have therefore identified a novel pathway that regulates Th2 immune responses in the gut that could potentially be targeted to upregulate host protective immune responses during gut parasite infection.

Recent data suggest that in certain chronic parasite infections, induction of Foxp3+ Tregs is important in suppression of protective immunity and development of chronic infection [30], [31], [32]. Given the fundamental role of TGFβ in induction of Foxp3+ Tregs from naive CD4+ T-cells, and the fact that *Itgb8* (*CD11c-Cre*) mice have previously been shown to have impaired induction of intestinal Foxp3+ Tregs [12], we hypothesised that protection from *T. muris* infection observed in *Itgb8* (*CD11c-Cre*) mice was due to reduced induction of Foxp3+ Tregs. However, when Foxp3+ Tregs were depleted before and during the course of infection no protection from infection was observed. Indeed, in contrast to *Itgb8* (*CD11c-Cre*) mice, no enhancement of CD4+ T-cell IL-13 production was observed early during infection in Foxp3+ Treg-depleted mice (Figure S7 in Text S1). Additionally, in agreement with previous reports [23] we did not see a significant increase in Foxp3+ Tregs during *T. muris* infection.

Instead, TGFβ activation by DC-expressed integrin αvβ8 appears important in suppression of IL-13 production by CD4+ T-cells early during *T. muris* infection. This is in agreement with previous data from *in vitro* studies, suggesting that TGFβ can downregulate expression of GATA-3 in T-cells (a key transcription factor in promoting Th2 cell differentiation) [33], [34]. Indeed, recent data suggest that TGFβ-mediated induction of the transcription factor Sox4 is important in preventing GATA-3 transcription to drive Th2 development [35]. Furthermore, we only observed an early increase in CD4+ T-cell specific pSmad2/3 signalling during a chronic Th1-promoting low dose infection and not during an acute Th2 promoting high dose infection in C57BL/6 mice (Figure S8 in Text S1) Thus, our data suggests that activation of TGFβ by integrin αvβ8 early during *T. muris* infection is important in suppression of protective Th2 cell development, which leads instead to production of an inappropriate Th1 response and development of chronic infection. Although we did not detect any IL-4 production in *Itgb8* (*CD11c-Cre*) mice during infection, given that we crossed these mice to IL4 KO mice to eliminate enhanced IL-13 production by T-cells, we cannot rule out a potential role for low level production of IL-4 (below our limits of detection) in protection from infection.

In addition to effects on T-cells, TGFβ has wide-ranging effects on multiple other immune cell types [36]. Recent reports have highlighted an important role for novel innate lymphoid cells in promoting protective type 2 immunity during certain parasite infections [18], [19], [20], [21]. Hence, it could be postulated that protection from infection seen in the absence of integrin αvβ8 results from an enhanced innate lymphoid cell response. However, protection from chronic *T. muris* infection observed in *Itgb8* (*CD11c-Cre*) mice did not correlate with enhanced type 2 cytokine production from any cell types apart from CD4+ T-cells (data not shown), and protection from infection was completely dependent on CD4+ T-cells. Thus, although innate lymphoid cell depletion would be required to definitively rule out their role in this enhanced Th2 response, it appears unlikely that lack of integrin-mediated TGFβ activation by DCs is promoting expulsion of the parasite via effects on non-CD4+ T-cells.

Given the crucial importance of TGFβ in regulating CD4+ T-cell responses, our current model is that TGFβ activated by DCs acts directly in CD4+ T-cells to regulate type 2 responses during *T. muris* infection. A recent study by Heitmann *et al.* (2012) suggests that CD4+ T-cell type 2 responses can be regulated via TGFβ signalling in DCs [36]. Thus, mice expressing a dominant negative construct of the TGFβ receptor II in myeloid cells (hence are refractory to TGFβ signalling) display enhanced Th2 responses during infection with the helminth *Nippostrongylus brasiliensis* [36]. However, we observed no difference in pSmad2/3 induction in DCs from control versus *Itgb8* (*CD11c-Cre*) mice early during infection (Figure S2 in Text S1). Thus, our data indicate that activation of TGFβ by integrin αvβ8 on DCs does not regulate Th2 cells indirectly via autocrine TGFβ signalling during *T. muris* infection.

Velhoden *et al* 2008 [37] have demonstrated that mice expressing a dominant negative TGFβ receptor specifically on CD4+ T-cells (CD4-DN-TGFβRII mice, thus T-cells are refractory to TGFβ) are more susceptible to infection with *T. muris* using an acute model of infection. This finding initially appears to conflict with our data, as we demonstrate that both antibody-mediated blockade of TGFβ and lack of the TGFβ activating integrin αvβ8 on DCs promotes expulsion of the parasite. However, recent data suggests that CD4-DN-TGFβRII mice display high levels of IFNγ level during intestinal helminth infection [38],[39] which, given the known role of IFNγ in promoting chronic *T. muris* infection [13], may explain the enhanced levels of infection observed in CD4-DN-TGFβRII mice.

An important question that remains are which specific subset of intestinal DCs are involved in modulating CD4+ T-cells to suppress Th2 responses? Although a functionally important gut population of CD11c+ T-cells does exist [16], which may be targeted in our CD11c-cre knock-out system, mice lacking integrin αvβ8 on T-cells (*Itgb8* (*CD4-Cre*) mice) were completely susceptible to *T. muris* infection (Figure S3 in Text S1). These data indicate that it is indeed an αvβ8-expressing DC population (or a related CD11c+ mononuclear phagocyte population) that is important in inhibiting Th2 responses in this infection. An important DC subset likely involved during infection are the migratory CD103+ DC [40], as we have previously demonstrated that this cell subset expresses high levels of integrin αvβ8 [12]. We have observed some integrin αvβ8 expression on the CD11c+ CD103- DC subset in the colon [12], which has been suggested to include both DCs and macrophage-like cell populations [41]. However, although some subsets of CD11c+ CD103- intestinal cells have been shown to migrate to lymph nodes to modulate T-cell responses [42], a large population do not normally migrate. Of note, we did not observe any alteration in the levels of αvβ8 expression on either CD103+ or CD103- LILP subset during the development of a chronic infection (Figure S9 in Text S1). Therefore, the exact DC population involved in downregulation of Th2 responses via integrin αvβ8 remains to be determined. Nevertheless, this key role for DC-expressed integrin αvβ8 in modulating Th2 responses, in addition to its previous essential roles in the induction of Foxp3+ Tregs [12] and Th17 cells [43], places DC-expressed integrin αvβ8 as a key orchestrator of CD4+ T-cell immunity.

In summary, we have highlighted an important cellular and molecular pathway by which the TGFβ-activating integrin αvβ8 expressed by DCs represses protective Th2 immunity during intestinal parasite infection with *T. muris*. Thus, given the poor treatments currently available for chronic parasite infection, further work should focus on the potential for targeting integrin αvβ8 therapeutically to enhance protective immunity during *Trichuris* infection. Additionally, whether the pathway is involved in the development of other chronic infections and Th2-associated disease is the focus of current studies.

## Materials and Methods

### Animals

C57 BL/6 mice were purchased from Harlan Laboratories. Mice lacking integrin αvβ8 on DCs via expression of a conditional floxed allele of β8 integrin in combination with CD11c-Cre (*Itgb8 (CD11c-Cre)* mice) [11], DEREG mice [24], GFP-Foxp3 mice [25] and IL-4−/− mice [44], all on a C57BL/6 background, have been previously described. All mice were maintained in specific pathogen-free conditions at the University of Manchester and used at 6 to 8 weeks of age.

### Ethics statement

All animal experiments were performed under the regulations of the Home Office Scientific Procedures Act (1986), specifically under the project licence PPL 40/3633. The project licence was approved by both the Home Office and the local ethics committee of the University of Manchester.

### *Trichuris muris* infection

The techniques used for maintenance and infection of *T. muris* were as previously described [45] Mice were orally infected with 20–30 eggs for a low-dose infection and 150 for an acute infection. Worm burdens were assessed by counting the number of worms present in the caecum as described previously [45].

### Antibody-mediated TGFβ blockade and cell depletion

To block TGFβ, mice were injected i.p with 0.5 mg of anti-TGFβ blocking antibody (clone 1d11.16.8) (BioXCell, NH, USA) or control IgG1 every 2 days from 4 days prior to infection. CD4+ cells were depleted via i.p. injection of 2 mg anti-CD4 antibody (YTS 191)^47^ every 2 days from 6 days prior to infection. Foxp3+ Tregs were depleted in DEREG mice as described [24], via i.p. injection of 200 ng diphtheria toxin (Merck) every 2 days from 2 days prior to infection.

### Adoptive transfer of Foxp3+ Tregs

Spleens were removed from Foxp3^GFP^ mice, disaggregated and red blood cells lysed. Cells were blocked with anti-FcγR antibody and labelled with anti-CD4 antibody (clone GK1.5; eBioscience) before sorting for CD4+, GFP+ populations using a FACS Aria. Cell purity in all experiments was >99.8%. Mice were injected i.p. with 0.5×10^6^ cells 3 days prior to infection.

### DC purification

mLNs were excised from mice and incubated with shaking for 20 min at 37°C in RPMI with 0.08 U/ml liberase blendzyme 3 (Roche) or 1 mg/ml collagenase VIII and 50 U/ml DNAseI, respectively. Cells were blocked with anti-FcγR antibody (clone 24G2; eBioscience) before enrichment using a CD11c enrichment kit and LS MACS column (Miltenyi Biotec). Enriched DCs were labelled with anti-CD11c antibody (clone N418; eBioscience) and sorted using a FACS Aria. In all experiments, subset purity was >95%.

### TGFβ activation assay

DCs were incubated with mink lung epithelial cells transfected with a plasmid containing firefly luciferase cDNA downstream of a TGFβ-sensitive promoter [15] in the presence of 1 µg/ml lipopolysaccharide. Co-cultures were incubated overnight in the presence of 80 µg/ml control mIgG or anti-TGFβ antibody (clone 1d11) and luciferase activity detected via the Luciferase Assay System (Promega) according to manufacturer's protocol. TGFβ activity was determined as the difference in luciferase activity between control mIgG-treated samples and samples treated with anti-TGFβ antibody.

### Flow cytometry staining

Mesenteric lymph nodes (mLNs) were removed from mice and disaggregated through a 100 µm sieve. Caecum and proximal colon were excised and lamina propria lymphocytes were prepared essentially as described [46] with slight modification in the tissue digestion step (digestion medium used was RPMI with 10% Foetal calf serum, 0.1% w/v collagenase type I and Dispase II (both Invitrogen), and tissue was digested for 30 min at 37°C). Cell suspensions were blocked with anti-FcγR antibody (clone 24G2; eBioscience) before labelling with antibodies specific for CD4 (clone GK1.5; eBioscience), Foxp3 (clone FJK-16s; eBioscience), IL-13 (clone eBiol13A; eBioscience), IFNγ (clone XMG1.2; eBioscience) or p-Smad 2/3 (Santa Cruz). For pSmad2/3 staining, an Alexa Fluor 594-labelled donkey anti-goat secondary antibody was used (Invitrogen). All samples were analysed on a FACS LSRII.

### Cell re-stimulation

mLN and LILP cells were prepared as described above before incubating with 50 ug/ml of concavelin A or *T. muris* excretory/secretory (E/S) antigen for 48 hours. Cell-free supernatants were analysed for cytokine production via cytometric bead array (BD) or paired ELISA antibodies (anti- IFNγ, clone XMG1.2 and biotin anti- IFNγ, clone R4-6A2; anti-IL-13, clone eBio13A and biotin anti-IL-13, clone eBio1316H and anti-IL-4, clone 11B1and biotin anti-IL-4, clone BVD6-2462 (eBioscience). For intracellular cytokine analysis cells were incubated for 12 hours with 50 ug/ml *T. muris* E/S antigen followed by PMA/ionomycin stimulation for 4 hour with addition of monensin for the final 3 hours. Cells were then stained with antibodies against IL-4, IL-13 and IFNγ using the eBioscience Foxp3 permibilization kit according to the manufacturer's instructions.

### Western blot

CD4+ T-cells were isolated from mLN via negative selection using a CD4+ T-cell isolation kit (Miltenyi Biotec) during the course of a chronic *T. muris* infection and lysed using 1% Triton-X100 in Tris buffer (50 mM Tris-HCl, 150 mM NaCl pH 7.4) plus 5 mM EDTA, 20 µg/ml leupeptin and aprotinin, 0.5 mM AESF and 2 mM NaVO_3_. Lysates were analysed by Western blot with antibodies to detect p-Smad 2/3 (Millipore) and β-actin (Sigma Aldrich), using the Invitrogen Nupage gel system according to manufacturer's instructions.

### Quantitative PCR

Total RNA was purified from sorted DC subsets using an RNAeasy minikit according to manufacturer's protocol (Qiagen). RNA was reverse transcribed using Oligo dT primers, and cDNA for specific genes detected using a SYBR green qPCR kit (Finnzymes) Gene expression normalised to HPRT expression. HPRT *Forward:* GCGTCGTGATTAGCGATGATGAAC, HPRT *Reverse:* GAGCAAGTCTTTCAGTCCTGTCCA, Integrin β8 *Forward:* GGGTGTGGAAACGTGACAAGCAAT, Integrin β8 *Reverse:* TCTGTGGTTCTCACACTGGCAACT.

### Statistical analysis

Results are expressed as mean ± SEM. Where statistics are quoted, 2 experimental groups were compared using the Student's *t*-test for non-parametric data. Three or more groups were compared using the Kruskal–Wallis test, with Dunn's multiple comparison post-test. *P*≤0.05 was considered statistically significant.

## Supporting Information

Text S1 **Supporting information associated with Worthington** ***et al*** **, 2013, containing Supplementary figures S1–S9.**
(PDF)
