# Supplementary material for: Loss of the TGFβ-Activating Integrin αvβ8 on Dendritic Cells Protects Mice from Chronic Intestinal Parasitic Infection via Control of Type 2 Immunity
Source: PLoS Pathog. 2013 Oct 3;9(10):e1003675. doi: 10.1371/journal.ppat.1003675 (PMC3789784; doi:10.1371/journal.ppat.1003675)
Supplement: Text S1 — Supporting information associated with Worthington et al , 2013, containing Supplementary figures S1–S9. (PDF) [file ppat.1003675.s001.pdf]

Supplementary figure legends

Figure S1. pSmad2/3 expression in LILP CD4+ T-cells during the development of a chronic *T. muris* infection. Representative flow cytometry plots for p-Smad 2/3 staining in CD4+ T-cells from control and *Itgb8 (CD11c-Cre)* mice at different times after infection of mice with a chronic dose of *T. muris* eggs. Representative of data from three independent experiments performed.

Figure S2. pSmad2/3 expression is not altered in DCs early during the development of chronic *T. muris* infection. (*A*) Representative flow cytometry plot and (*B*) pooled data for expression of p-Smad 2/3 in mLN DCs at day 3 post-infection (p.i.) in control and *Itgb8 (CD11c-Cre)* mice after infection with a chronic dose of *T. muris* eggs. Data (n= 2–3 mice per group) are from at least two independent experiments performed. N.S., not significant via Student's *t*-test for the indicated comparisons between groups, error bars represent SE of means.

Figure S3. Mice lacking the TGFβ-activating integrin αvβ8 on CD4+ cells are susceptible to a chronic *T. muris* infection. (*A*) Worm burdens from control and *Itgb8 (CD4-Cre)* mice were analysed at day 35 post-infection (p.i.) with a chronic dose of *T. muris* eggs. (*B*) Parasite-specific serum IgG1 and IgG2a levels in control and *Itgb8 (CD4-Cre)* mice at day 35 p.i. with a chronic dose of *T. muris* eggs via ELISA (n= 4-5 mice per group) are from two independent experiments. N.S., not significant Student's *t*-test for the indicated comparisons between groups, error bars represent SE of means.

Figure S4. Successful ablation of CD4+ T-cells from *Itgb8 (CD11c-Cre)* mice and GFP-Foxp3+ Tregs from DEREG mice. (*A*) Percentage of CD4+ T-cells from spleen or mLN of *Itgb8 (CD11c-Cre)* mice treated with 2mg of control IgG or anti-CD4 (YTS191) antibody every 2 days were analysed by flow cytometry 17 days post-infection (p.i.) with a chronic dose of *T. muris* eggs. Data (n= 6 mice per group) are from two independent experiments performed. (*B*) The percentage of GFP-Foxp3+ Tregs of CD4+ T-cells in spleen and mLN was analysed in DEREG mice, treated with P.B.S. or 200ng diphtheria toxin every 2 days, at days 14 p.i. with a chronic dose of *T*. *muris* eggs. Data (n=5–10 mice per group) are from two independent experiments performed. Representative flow cytometry plots and data from individual mice are shown. ***, P<0.005 via Student's *t*-test for the indicated comparisons between groups; error bars represent SE of means.

Figure S5. Adoptive transfer of GFP-Foxp3+ Tregs restores Treg levels in *Itgb8 (CD11c-Cre)* mice. Caecal lamina propria Tregs were assessed in control, *Itgb8 (CD11c-Cre)* and *Itgb8 (CD11c-Cre)* mice injected with 0.5×10^6 GFP-Foxp3+ CD4 T-cells 3 days prior to infection. All mice received a chronic dose of *T*. *muris* and were analysed at day 3 post-infection. Representative flow cytometry plots shown.

Figure S6. Mice lacking the TGFβ-activating integrin αvβ8 on DCs demonstrate no detectable IL-4 production early during chronic *T. muris* infection. IL-4 cytokine levels from ConA-stimulated mLN and LILP cells from control and *Itgb8 (CD11c-Cre)*

mice were analysed at different time-points post-infection (p.i.) with a chronic dose of *T*. *muris* eggs. Cytokine levels were determined via cytometric bead array/ELISA. Data (n= 3–10 mice per group) are from two or more independent experiments. N.D., not detectable; N.S., not significant via Student's *t*-test for the indicated comparisons between groups, error bars represent SE of means.

Figure S7. Depletion of Foxp3+ Tregs during development of chronic *T. muris* infection does not result in enhanced IL-13 production in CD4+ T-cells. Representative flow cytometry plots and pooled data for percentage of intracellular IL-13+ cells of LILP CD4+ T-cells at day 3 post-infection (p.i.) with a chronic dose of *T*. *muris* eggs in control and DEREG mice, following treatment with 200ng diphtheria toxin 2 days before, on the day of infection and 2 days p.i.. Data (n=4-6 mice per group) are from two independent experiments performed. N.S., not significant via Student's *t*-test for the indicated comparisons between groups, error bars represent SE of means.

Figure S8. pSmad2/3 expression in CD4+ T-cells during the development of chronic and acute *T. muris* infection. Representative flow cytometry plots for expression of p-Smad 2/3 in mLN CD4+ T-cells in naive and day 3 and 7 post-infection (p.i.) in control and *Itgb8 (CD11c-Cre)* mice after infection with a chronic or acute dose of *T*. *muris* eggs. Representative of data from three independent experiments performed.

Figure S9. Intestinal DC expression of the TGFβ-activating integrin αvβ8 is not altered during development of a chronic *T. muris* infection. RNA was purified from CD103+/- DCs from LILP and mLN from naive and day 3 and 7 post-infection (p.i.) C57BL6 mice, after infection with a chronic dose of *T. muris* eggs, and analysed for integrin β8 expression by quantitative RT-PCR. Data is normalized to CD103- mLN integrin β8 expression. Representative of data from two independent experiments performed. N.S., not significant via Kruskal–Wallis for the indicated comparisons between groups, error bars represent SE of means.

FIGURE S1

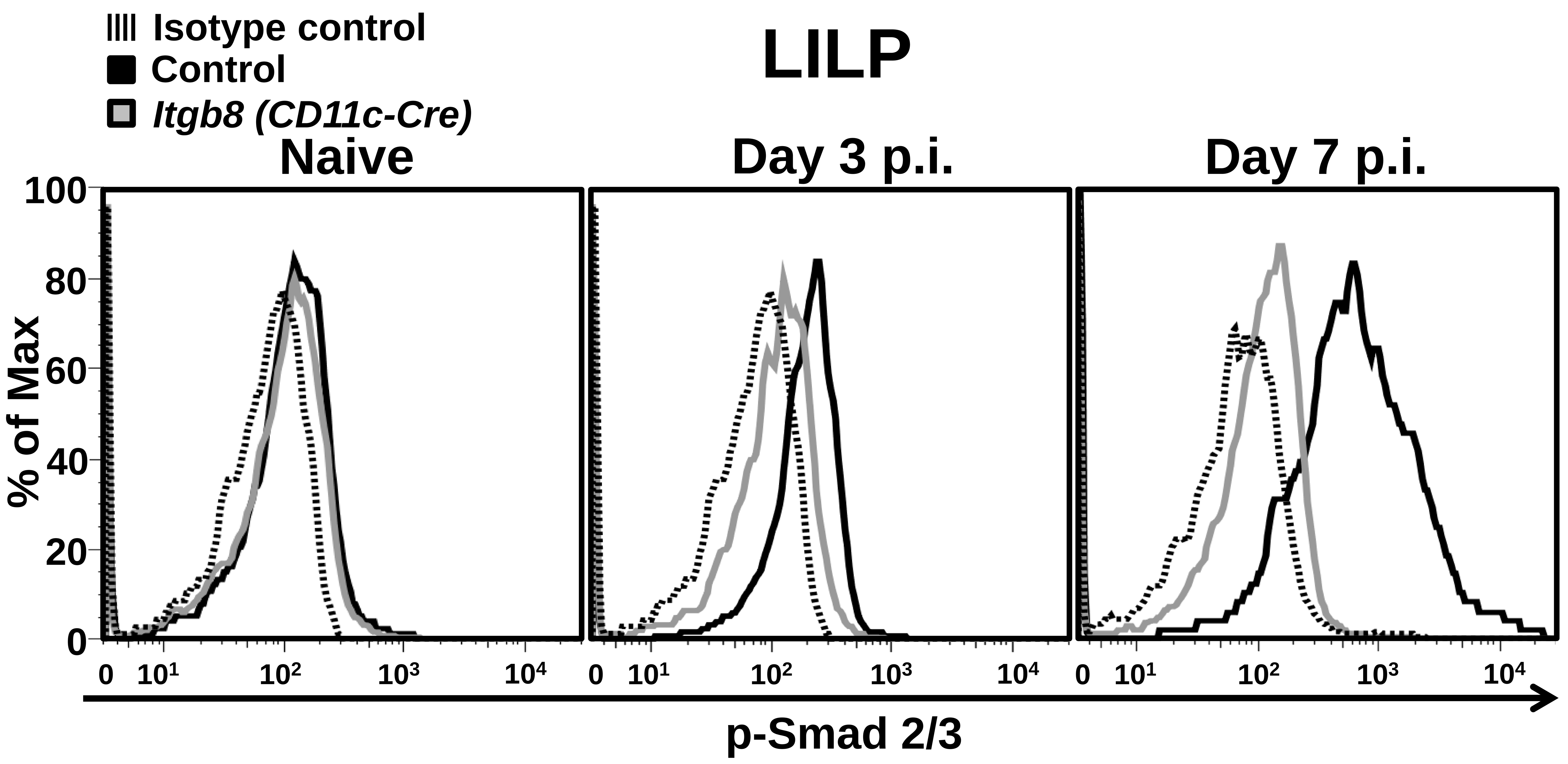
Isotype control
Control
Itgb8 (CD11c-Cre)
LILP
Naive
Day 3 p.i.
Day 7 p.i.
100
80
60
40
20
0
% of Max
0
10¹
10²
10³
10⁴
p-Smad 2/3

FIGURE S2

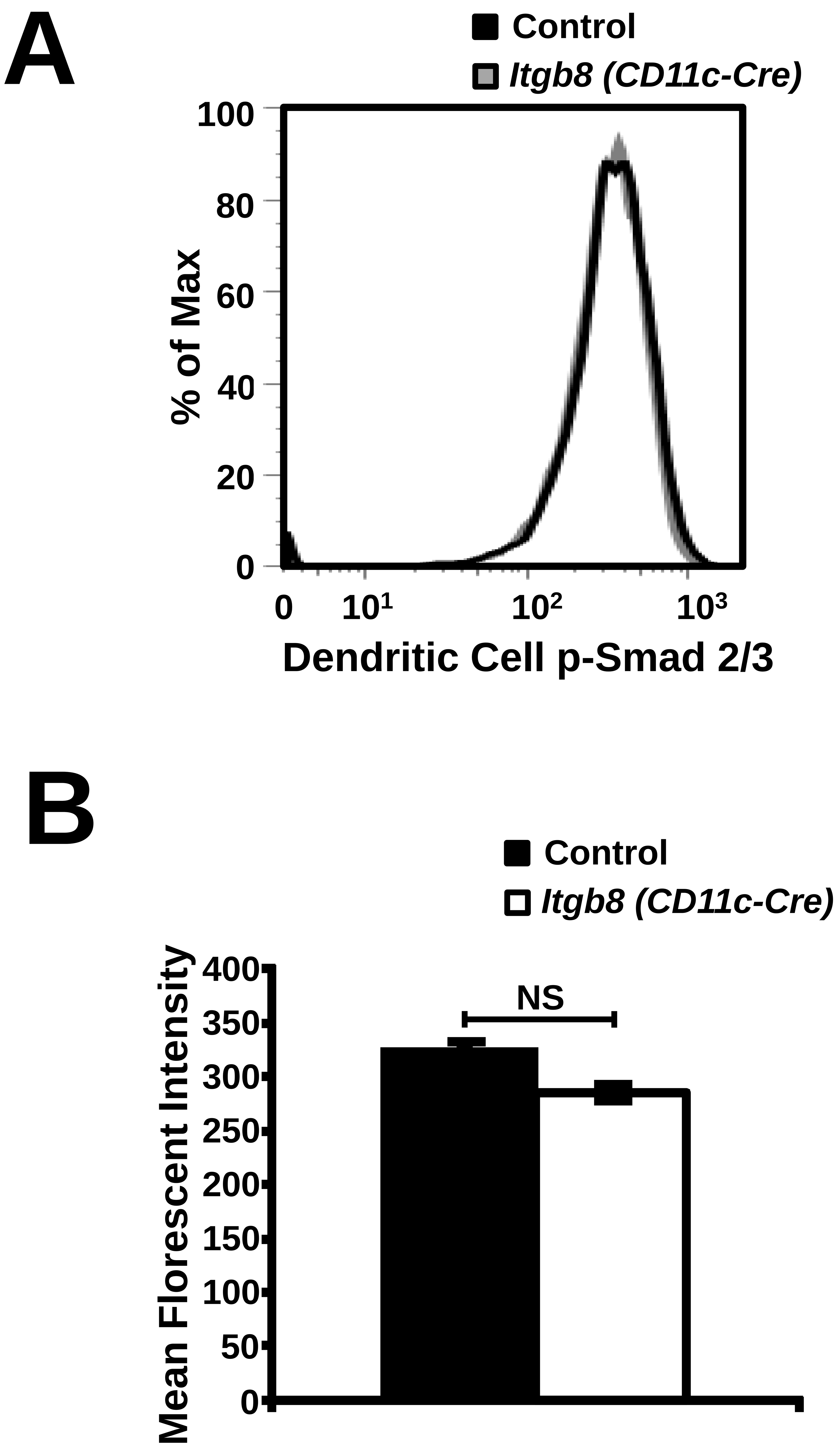
A
Control
Itgb8 (CD11c-Cre)
100
80
60
40
20
0
% of Max
0
10¹
10²
10³
Dendritic Cell p-Smad 2/3
B
Control
Itgb8 (CD11c-Cre)
NS
400
350
300
250
200
150
100
50
0
Mean Florescent Intensity

FIGURE S3

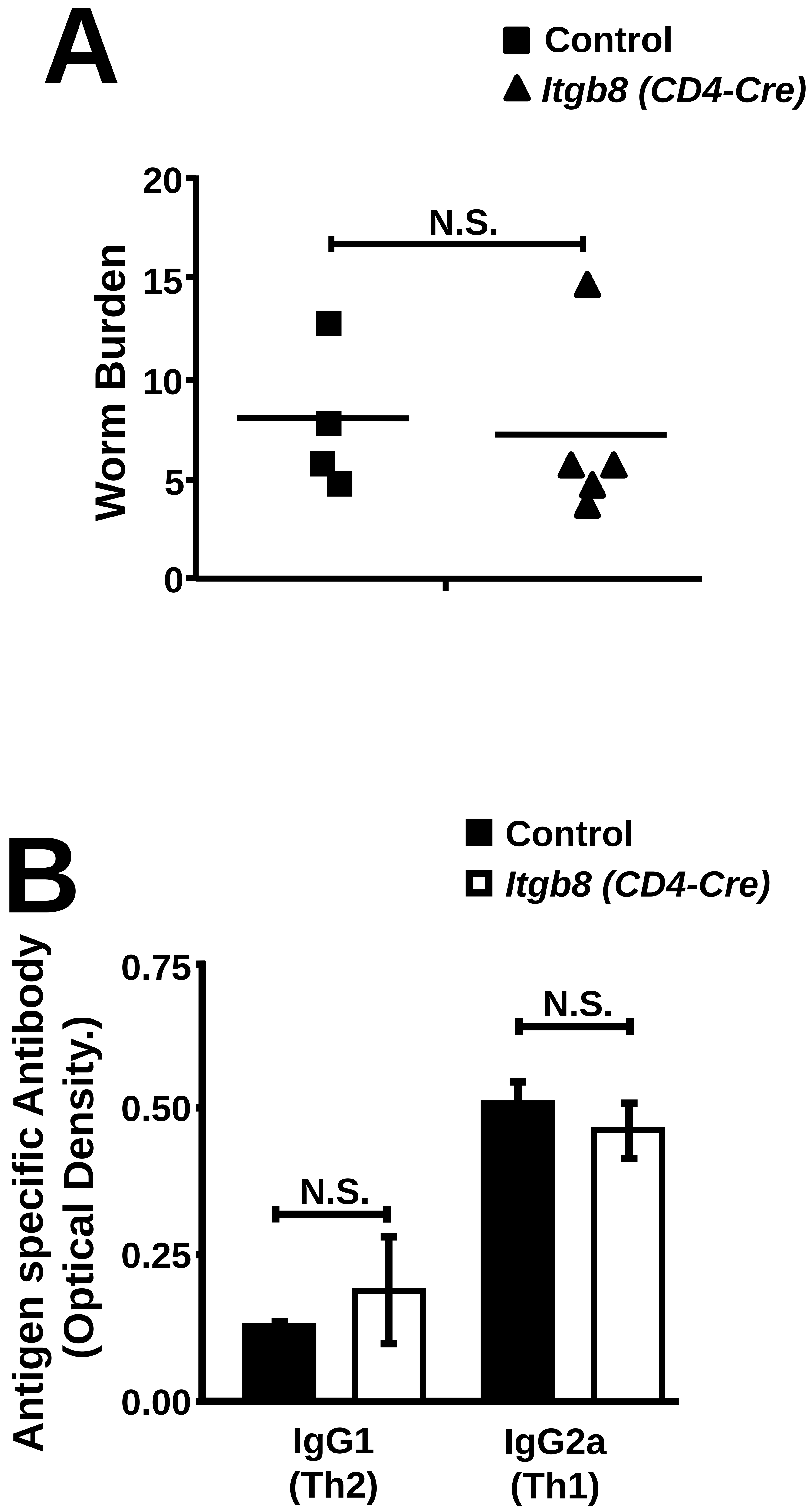
A
Control
Itgb8 (CD4-Cre)
Worm Burden
N.S.
20
15
10
5
0
B
Control
Itgb8 (CD4-Cre)
Antigen specific Antibody
(Optical Density.)
0.75
0.50
0.25
0.00
N.S.
N.S.
IgG1
(Th2)
IgG2a
(Th1)

FIGURE S4

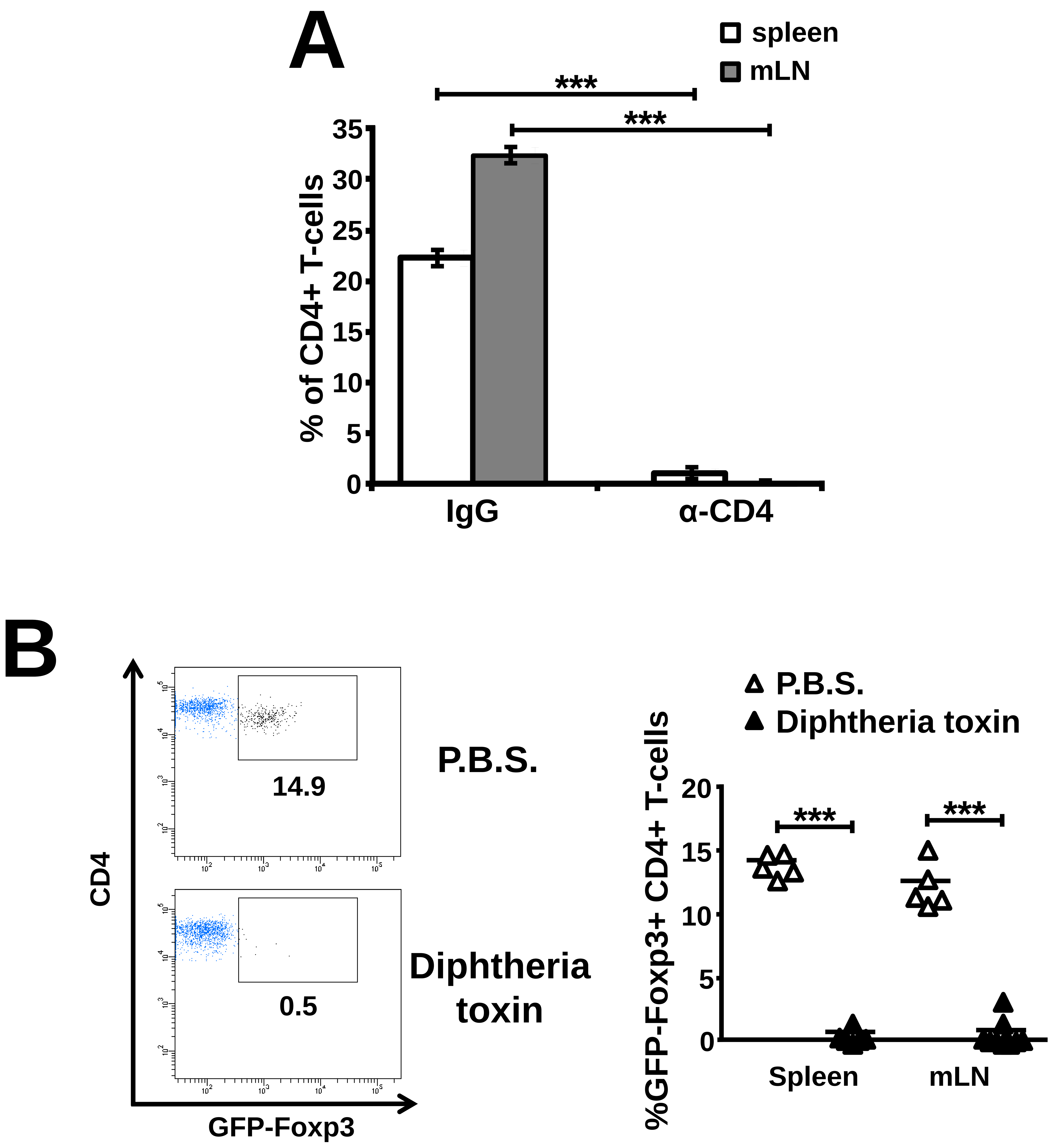
A
spleen
mLN
***
***
% of CD4+ T-cells
35
30
25
20
15
10
5
0
IgG
α-CD4
B
CD4
GFP-Foxp3
14.9
P.B.S.
0.5
Diphtheria toxin
P.B.S.
Diphtheria toxin
%GFP-Foxp3+ CD4+ T-cells
20
15
10
5
0
***
***
Spleen
mLN

FIGURE S5

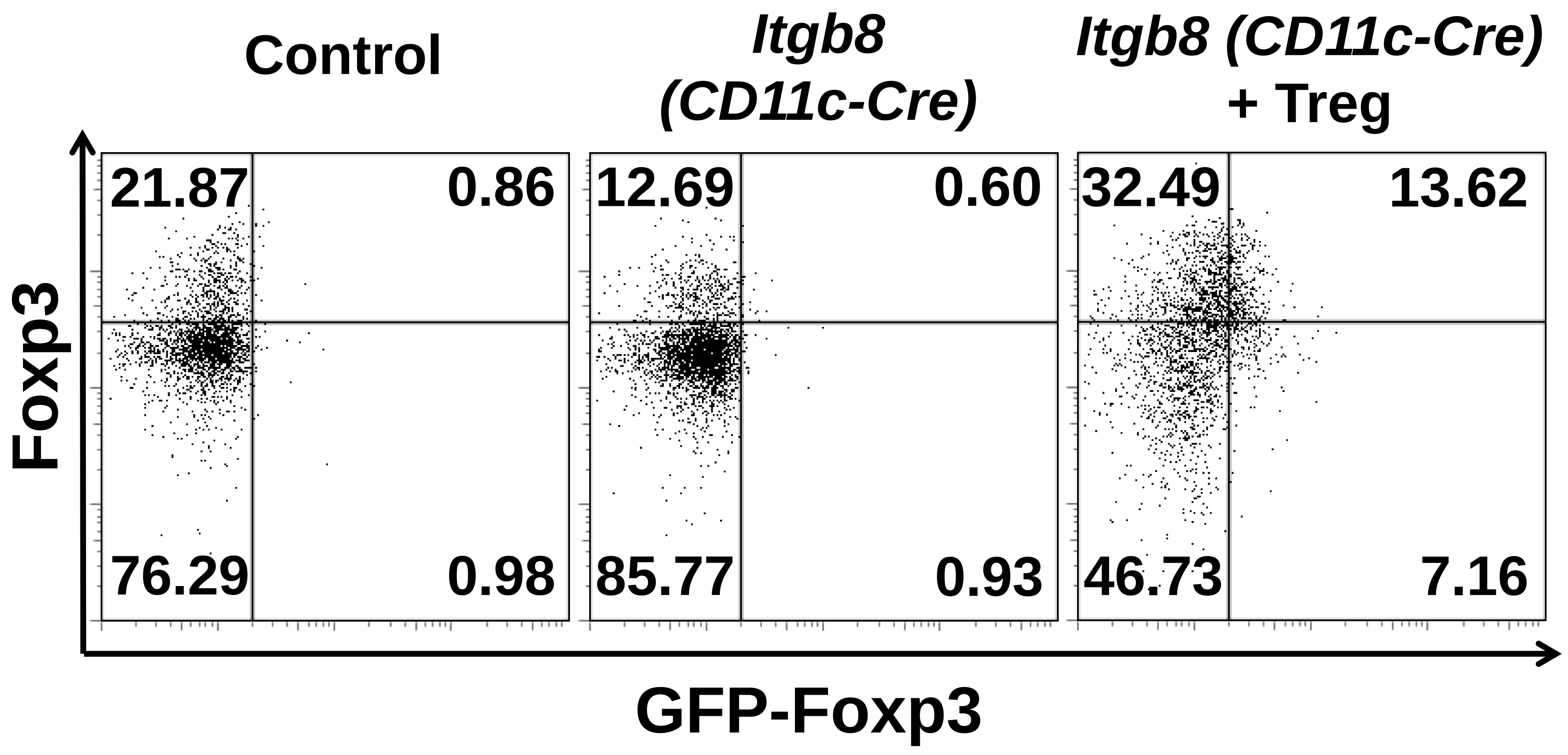

Control
Itgb8 (CD11c-Cre)
Itgb8 (CD11c-Cre) + Treg
Foxp3
GFP-Foxp3
21.87
0.86
76.29
0.98
12.69
0.60
85.77
0.93
32.49
13.62
46.73
7.16

FIGURE S6

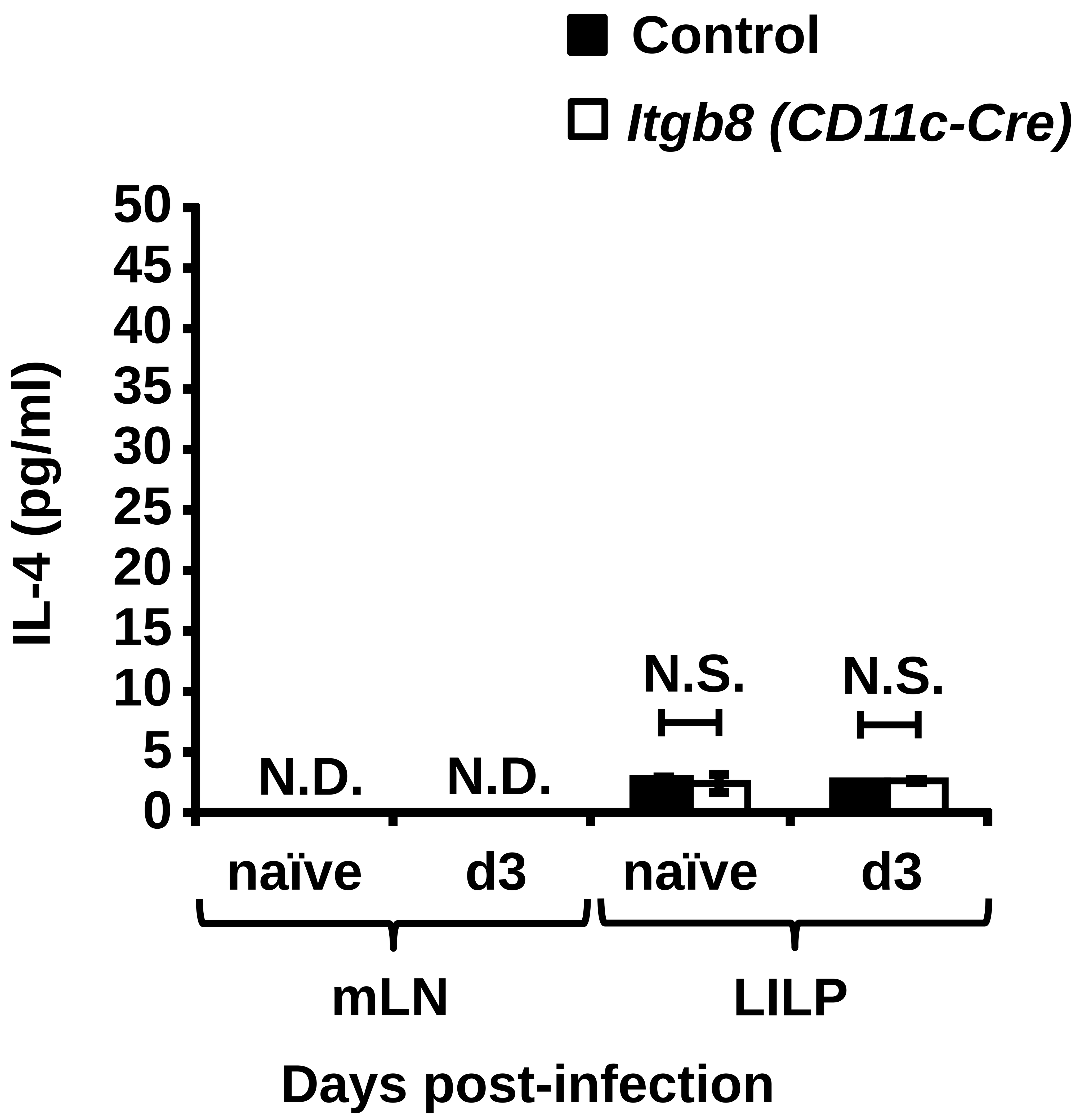
Control
Itgb8 (CD11c-Cre)
IL-4 (pg/ml)
50
45
40
35
30
25
20
15
10
5
0
N.D.
N.D.
N.S.
N.S.
naïve
d3
naïve
d3
mLN
LILP
Days post-infection

FIGURE S7

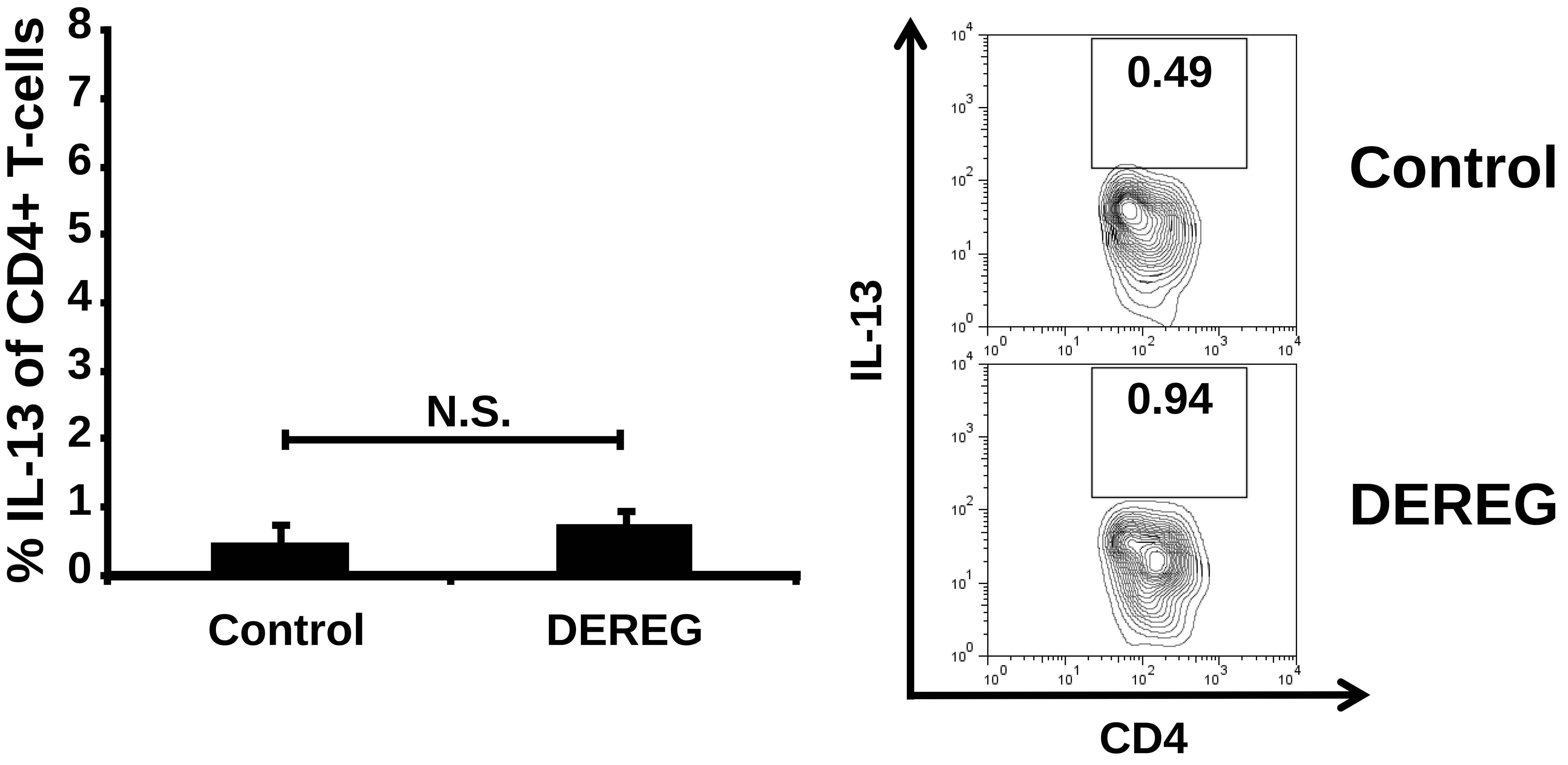

% IL-13 of CD4+ T-cells
8
7
6
5
4
3
2
1
0
N.S.
Control
DEREG
IL-13
0.49
Control
0.94
DEREG
CD4

FIGURE S8

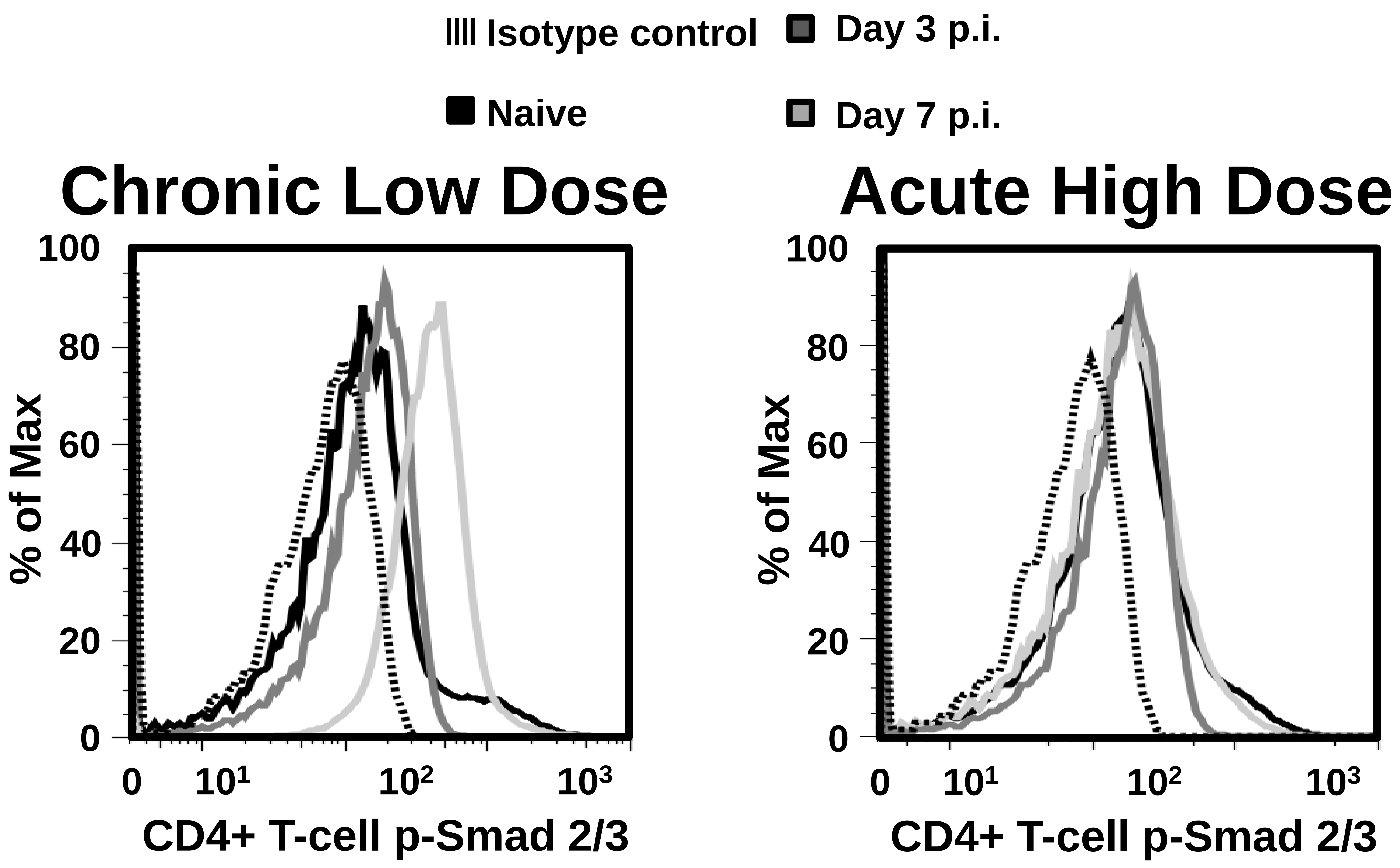

Isotype control
Day 3 p.i.
Naive
Day 7 p.i.
Chronic Low Dose
Acute High Dose
100
80
60
40
20
0
% of Max
0
10¹
10²
10³
CD4+ T-cell p-Smad 2/3

FIGURE S9

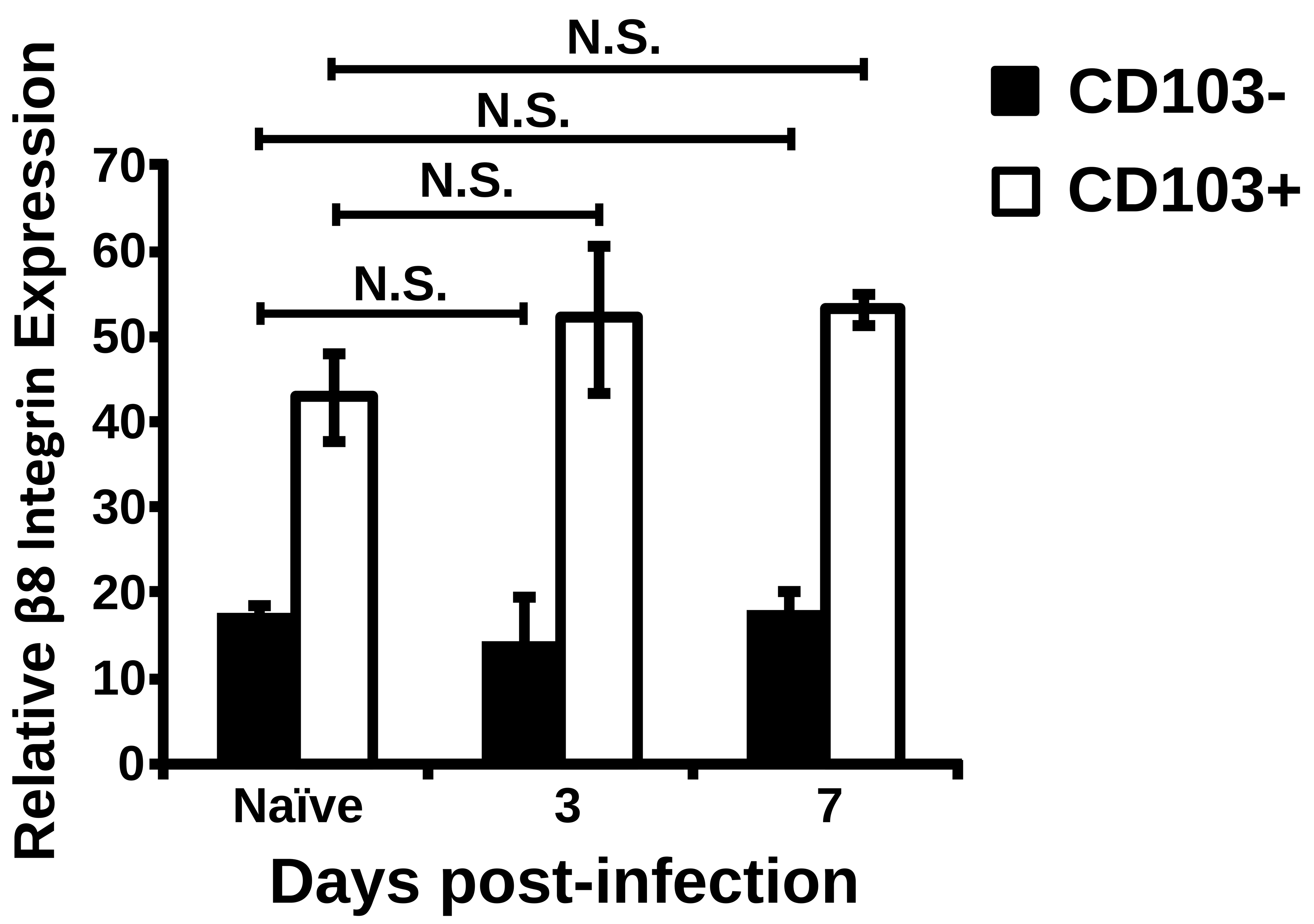
N.S.
N.S.
N.S.
N.S.
CD103-
CD103+
Relative β8 Integrin Expression
70
60
50
40
30
20
10
0
Naïve
3
7
Days post-infection
